# Gender Differences in X (Formerly Twitter) Use, Influence, and Engagement Among Cardiologists From the Top U.S. News Best Hospitals

**DOI:** 10.2196/66308

**Published:** 2025-06-04

**Authors:** Minji Seok, Sungjin Kim, Harper Tzou, Olivia Peony, Mitchell Kamrava, Andriana P Nikolova, Katelyn M Atkins

**Affiliations:** 1 Department of Medicine, Cedars-Sinai Medical Center, Los Angeles, CA, United States; 2 Biostatistics Research Center, Cedars-Sinai Medical Center, Los Angeles, CA, United States; 3 Department of Radiation Oncology, Cedars-Sinai Medical Center, 8700 Beverly Blvd AC1019, Los Angeles, CA, 90048, United States, 1 310-423-2178, 1 310-659-3332; 4 Department of Cardiology, Cedars-Sinai Medical Center, Los Angeles, CA, United States

**Keywords:** social media, women in medicine, health communication, technology, Twitter, cardiologist, cardiology, US, United States, heart, vascular surgery, adult, women, female, medicine

## Introduction

Women in medicine face significant barriers to compensation, career advancement, and research support, even when controlling for specialty, age, and/or clinical experience [1]. These barriers are especially pronounced in cardiology, where women comprise only 15% of practicing cardiologists and are less likely to be clinical trial leaders or present late-breaking trials at major cardiovascular conferences [2-4]. Social media platforms, such as X (formerly Twitter), can foster collaboration, mentorship, and promotion of research [5,6]. However, studies examining X’s impact on existing gender gaps are limited. In this study, we aimed to analyze differences between X users and non–X users and differences in X use by gender among adult cardiologists.

## Methods

### Ethical Considerations

This cross-sectional study was exempt from ethical approval by the Cedars-Sinai institutional review board due to the use of publicly available data.

### Study Design

The top 20 U.S. News Best Hospitals for cardiology, heart surgery, and vascular surgery were identified from the 2023 ranking (Table 1) [7]. Available physician website profiles of fellowship-trained adult medicine cardiologists were manually reviewed by 3 investigators (MS, HT, and OP) for inclusion, and demographic information was collected (eg, academic appointment, apparent gender, and medical school and fellowship graduation years). Physicians were evaluated for the presence of an X account, and public data were manually collected between December 8, 2023, and May 9, 2024. Differences between non–X users and X users and between women and men X users were compared, using Wilcoxon rank-sum tests for continuous variables and chi-square or Fisher exact tests for categorical variables as appropriate.

**Table 1.** Top 20 U.S. News Best Hospitals for cardiology, heart surgery, and vascular surgery (2023 ranking).

Institution name	State	Total physicians (N=2022), n (%)	Physicians on X (n=753), n (%)
Brigham and Womens	Massachusetts	143 (7.07)	73 (9.69)
Cedars Sinai	California	56 (2.77)	22 (2.92)
Cleveland Clinic	Florida and Ohio	126 (6.23)	51 (6.77)
Johns Hopkins	Maryland	102 (5.04)	35 (4.65)
Houston Methodist	Texas	64 (3.17)	33 (4.38)
Lenox Hill at Northwell	New York	117 (5.79)	27 (3.59)
Massachusetts General	Massachusetts	100 (4.95)	57 (7.57)
Mayo Clinic Rochester	Minnesota	156 (7.72)	68 (9.03)
Mount Sinai	Florida, New Jersey, and New York	201 (9.94)	64 (8.50)
NewYork-Presbyterian Hospital Columbia and Cornell	New York	54 (2.67)	15 (1.99)
NYU Langone Hospitals	New York	164 (8.11)	20 (2.66)
Northwell Northshore	New York	93 (4.60)	15 (1.99)
Northwestern	Illinois	112 (5.54)	47 (6.24)
Rush University	Illinois	44 (2.18)	22 (2.92)
Stanford Hospital	California	88 (4.35)	45 (5.98)
Texas Heart Institute at Baylor	Texas	14 (0.69)	5 (0.66)
University of California, Los Angeles	California	76 (3.76)	29 (3.85)
UT Southwestern	Texas	77 (3.81)	38 (5.05)
University of Pennsylvania	Pennsylvania	134 (6.63)	58 (7.70)
Vanderbilt	Tennessee	101 (5.00)	29 (3.85)

## Results

In total, 2022 cardiology physician profiles were analyzed; 37.61% (n=753) were on X, and 63.39% (n=1269) were not on X. Compared to nonusers, X users had a higher proportion of women (240/753, 31.87% vs 269/1269, 21.20%), higher academic faculty appointments, and a greater number of advanced degrees (all *P*<.001). Women and men X users had similar total practice durations (counted from fellowship training completion until 2024; median 10, IQR 1-45 y vs median 12, IQR 1-48 y; *P*=.14), but women’s practice durations since joining X were significantly lower (median 6.4, IQR 5-11 y vs median 7.8, IQR 5-10 y; *P*<.001). After adjusting for the number of years on X, women and men showed similar numbers of followers (median 71.46, IQR 24.8‐180.84 vs median 78.05, IQR 24.96‐197.33 per year on X; *P*=.68) and posts (median 29.1, IQR 5.06‐102.47 vs median 28.04, IQR 5.22‐111.15 per year on X; *P*=.98), but women had higher levels of self-engagement (number of users followed: median 42.11, IQR 16.8‐84.77 vs median 31.9, IQR 11.48‐70.4 per year on X; *P*=.02; number of liked posts: median 112.52, IQR 16.58‐430.1 vs median 64.49, IQR 6.94‐318.98 per year on X; *P*=.02; Table 2). Per a thematic analysis of biographical text, women were more likely than men to mention being a parent (48/239, 20.08% vs 64/513, 12.48%; *P*=.006), but there was no significant difference in mentions of jobs (*P*=.36) or hobbies (*P=*.89; Table 2).

**Table 2.** Characteristics and demographics of top hospital cardiologists on X, stratified by gender.

Variable	Not on X (n=1269)	On X (n=753)	*P* value^a^	Men on X (n=513)	Women on X (n=240)	*P* value^b^
Geographic region, n (%^c^)			<.001			.72
Northeast	741 (58.39)	364 (48.34)		245 (47.76)	119 (49.58)	
Midwest	249 (19.62)	187 (24.83)		130 (25.34)	57 (23.75)	
South	155 (12.21)	106 (14.08)		69 (13.45)	37 (15.42)	
West	124 (9.77)	96 (12.75)		69 (13.45)	27 (11.25)	
Gender, n (%^c^)			<.001			—^d^
Men	1000 (78.8)	513 (68.13)		—	—	
Women	269 (21.20)	240 (31.87)		—	—	
Faculty type, n (%^c^)			<.001			.06
Not explicitly listed	347 (27.34)	191 (25.37)		135 (26.32)	56 (23.33)	
Instructor/clinician	97 (7.64)	39 (5.18)		21 (4.09)	18 (7.5)	
Assistant	441 (34.75)	227 (30.15)		149 (29.04)	78 (32.5)	
Associate	208 (16.39)	153 (20.32)		100 (19.49)	53 (22.08)	
Professor	176 (13.87)	143 (18.99)		108 (21.05)	35 (14.58)	
Number of leadership titles, n (%^c^)			<.001			.11
0	840 (66.19)	360 (47.81)		239 (46.59)	121 (50.42)	
1	306 (24.11)	241 (32.01)		159 (30.99)	82 (34.17)	
2	95 (7.49)	111 (14.74)		85 (16.57)	26 (10.83)	
≥3	28 (2.21)	41 (5.44)		30 (5.85)	11 (4.58)	
Subspecialty, n (%^c^)			<.001			<.001
General	552 (43.53)	213 (28.29)		133 (25.93)	80 (33.33)	
Interventional	226 (17.82)	112 (14.87)		90 (17.54)	22 (9.17)	
Imaging	193 (15.22)	121 (16.07)		68 (13.26)	53 (22.08)	
Congenital	31 (2.44)	24 (3.19)		12 (2.34)	12 (5)	
Heart failure	91 (7.18)	121 (16.07)		78 (15.2)	43 (17.92)	
Electrophysiology	138 (10.88)	95 (12.62)		84 (16.37)	11 (4.58)	
Other	37 (2.92)	67 (8.9)		48 (9.36)	19 (7.92)	
Dual degree, n (%^c^)						
PhD			<.001			.34
No	1183 (93.22)	662 (87.92)		447 (87.13)	215 (89.58)	
Yes	86 (6.78)	91 (12.08)		66 (12.87)	25 (10.42)	
MS			<.001			.71
No	1220 (96.14)	679 (90.17)		464 (90.45)	215 (89.58)	
Yes	49 (3.86)	74 (9.83)		49 (9.55)	25 (10.42)	
MPH			<.001			.55
No	1226 (96.61)	680 (90.31)		461 (89.86)	219 (91.25)	
Yes	43 (3.39)	73 (9.69)		52 (10.14)	21 (8.75)	
MBA			.24			.76
No	1255 (98.9)	740 (98.27)		503 (98.05)	237 (98.75)	
Yes	14 (1.1)	13 (1.73)		10 (1.95)	3 (1.25)	
Practice duration (years)			<.001			.14
Median (IQR)	21 (12‐31)	11 (6‐21)		12 (1‐48)	10 (1‐45)	
Overall: <9; physicians on X: <7, n (%^e^)	152 (37.91)	249 (62.09)		117 (68.82)	53 (31.18)	
Overall: ≥9 and <17; physicians on X: ≥7 and <11, n (%^e^)	270 (60.81)	174 (39.19)		153 (63.22)	89 (36.78)	
Overall: ≥17 and <28; physicians on X: ≥11 and <21, n (%^e^)	292 (67.13)	143 (32.87)		123 (69.49)	54 (30.51)	
Overall: ≥28; physicians on X: ≥21, n (%^e^)	359 (80.86)	85 (19.14)		120 (73.17)	44 (26.83)	
X use variables (publicly available), median (IQR)						
Time on X (years)	—	—	—	7.80 (5.30‐11.34)	6.39 (5.06‐10.11)	<.001
Average number of followers per year on X	—	—	—	78.05 (24.96‐197.33)	71.46 (24.8‐180.84)	.68
Average number of people followed per year on X	—	—	—	31.90 (11.48‐70.40)	42.11 (16.8‐84.77)	.02
Average number of tweets per year on X	—	—	—	28.04 (5.22‐111.15)	29.10 (5.06‐102.47)	.98
Average number of media posts per year on X	—	—	—	2.27 (0.26‐10.38)	2.20 (0.26‐10.78)	.96
Average number of liked posts per year on X	—	—	—	64.49 (6.94‐318.98)	112.52 (16.58‐430.1)	.02
Thematic content of X biography, n (%^c^)						
Job Roles			—			.36
No mention	—	—		98 (19.10)	39 (16.32)	
Mention	—	—		415 (80.90)	200 (83.68)	
Specialty			—			.48
No mention	—	—		169 (32.94)	85 (35.56)	
Mention	—	—		344 (67.06)	154 (64.44)	
Parent			—			.006
No mention	—	—		449 (87.52)	191 (79.92)	
Mention	—	—		64 (12.48)	48 (20.08)	
Spouse			—			.77
No mention	—	—		467 (91.03)	216 (90.38)	
Mention	—	—		46 (8.97)	23 (9.62)	
Institution			—			.56
No mention	—	—		148 (28.85)	64 (26.78)	
Mention	—	—		365 (71.15)	175 (73.22)	
Personal interests			—			.89
No mention	—	—		444 (86.55)	206 (86.19)	
Mention	—	—		69 (13.45)	33 (13.81)	

a Not on X versus on X. The *P* values were calculated via Wilcoxon rank-sum tests for continuous and ordinal variables and via chi-square tests or Fisher exact tests for categorical variables, as appropriate.

b Men on X versus women on X. The *P* values were calculated via Wilcoxon rank-sum tests for continuous and ordinal variables and via chi-square tests or Fisher exact tests for categorical variables, as appropriate.

c Column %: these percentages were calculated based on the total n values for the columns of this section.

d Not applicable.

e Row %: these percentages were calculated based on the total n values for the rows of this section.

## Discussion

In our analysis of U.S. News Best Hospitals cardiologists, the proportion of women on X was higher than the proportion of women non–X users. One possible explanation for this is that women cardiologists may be seeking novel opportunities for networking, collaboration, visibility, and/or self-promotion that are not available through traditional channels [5]. Additionally, compared to men, women cardiologists had similar time-adjusted follower counts but liked more posts. This is consistent with content language analyses demonstrating higher expected levels of friendliness in women’s professional communications, including more frequent use of exclamation points as markers of friendly interaction, which is associated with increased emotional labor [8,9]. Further, women cardiologists were more likely to mention being a parent, suggesting that women may be more comfortable with highlighting work-life integration factors. This is unsurprising, as women physicians have joined social media groups discussing issues such as parenting, maternity leave, and women leadership in medicine [5]. These observations support efforts to better understand motivational differences in social media use and impacts on potential downstream professional benefits.

Our study has several limitations, including institutional websites being subject to inaccuracy and incompleteness, currently available X data being more limited compared to prior studies, limited physician practice type information, and potential misgendering [10]. However, our findings highlight the increased presence of women cardiologists on X, with similar influence to men and higher engagement despite shorter time on X. These findings suggest an inherent desire to engage on social media for professional use, though the motivating factors driving these behavioral differences and their impact on existing gender disparities warrant further study.
